# Reduced Expression of Galectin-8 May Contribute in Carcinogenic Pathway of Head and Neck Squamous Cell Carcinoma

**DOI:** 10.30699/IJP.2021.121140.2318

**Published:** 2021-03-02

**Authors:** Maryam Ghasemi, Laleh Vahedi Larijani, Jamshid Yazdani-Charati, Elham Kamali Hakim

**Affiliations:** 1 *Department of Pathology, Immunogenetics Research Center, School of Medicine, Mazandaran University of Medical Sciences, Sari, Iran*; 2 *Department of Biostatistics, Health Sciences Research Center, School of Health, Mazandaran University of Medical Sciences, Sari, Iran *; 3 *Department of Pathology, School of Medicine, Mazandaran University of Medical Sciences, Sari, Iran*

**Keywords:** Galectin-8 Head and Neck Squamous Cell Carcinoma Immunohistochemistry

## Abstract

**Background & Objective:**

Galectin-8 has relationships with cell growth and metastasis of some cancers. Due to controversy in the clinical significance of this protein in the cancer process, we investigated its roles in the development of head and neck squamous cell carcinoma.

**Methods:**

This study was performed on 93 samples of patients with Squamous Cell Carcinoma or dysplasia of the head and neck, who underwent biopsy or surgery from 2015 till 2017 in Boo-Ali SINA hospital of Sari, Iran. The relevant paraffin embedded tissue blocks were obtained from archive of pathology and evaluated for galectin-8 by immunohistochemistry. The association between expression of galactin-8 and age, sex, location and stage of disease were assessed. To compare expression rate between the groups, Mc-Nemar, Chi-square and Fisher's exact tests were used. The P-value<0.05 was considered significant.

**Results:**

Strong cytoplasmic and nuclear galactin-8 staining was observed in 97.6% cases of normal tissues while 77% of dysplastic lesions and 69% of the cancers revealed negative immunoreactivity. The intensity of expression in dysplastic and malignant tissues was significantly reduced compared with normal tissues (*P*=0.0001). The expression of galectin-8 did not correlate with stage (*P*=0.303), lymph node involvement (*P*=0.326), tumor grade (*P*=0.769), distant metastasis (*P*=0.748), and age (*P*=0.574).

**Conclusion:**

We observed that the expression of galectin-8 in dysplastic and malignant squamous epithelium significantly reduced compared with the normal counterpart of them in the head and neck. It may contribute to malignant transformation of head and neck squamous cells.

## Introduction

Galectins are a group of proteins that bind to beta-galactosidases in a cell through a specific sequence of carbohydrate recognition domain (CRD), and each galectin has its own CRD and can be seen in the cytoplasm or nucleus of the cell. Galectins are differently expressed in the various normal cells. They localize in the extracellular matrix, at surface of the cells, in the cell cytoplasm or nucleus correspondingly to biological effects of the cell (1-4).

In general, Galectins contain 15 types of proteins, which are divided into three major groups based on protein molecular structure. The first group is the prototype of galectin, which has a CRD. The second group is Tandem repeat galectin, which has two CRDs, and the third group is Chimera type galectin, which has a large N-terminal and is attached to a CRD (1,4-7). Galectin-8 (Gal-8) is a protein of 35 kDa, which belongs to a tandem repeat of structurally different carbohydrate recognition domains within a single polypeptide chain. It was previously isolated from a rat liver cDNA expression library and was found to be structurally homologous to rat Galectin- 4 (Gal-4) (34%) in the year of 1995 (2,5).

Immobilized Gal-8 functions as a matrix protein similar to fibronectin and promote cell adhesion and migration by ligation and clustering integrin receptors and triggers integrin-mediated signaling cascades such as Tyr phosphorylation of FAK and paxillin whereas soluble Gal-8 with interaction with cell surface integrin inhibit cell adhesion. Similarly, Gal-8 interacting with cell surface ligands inﬂuences T cell proliferation, induces apoptosis, modulates neutrophil function, and promotes cell spreading and cytoskeletal arrangement (5-8). 

Gal-8 is expressed extensively in the normal tissue, for example, in the gastrointestinal (GI) system especially in the cytoplasm as well as in the nucleus of the cells in the normal tissue of the colon. However, in colon cancer, it is almost always expressed in the cytoplasm. Studies have shown that in colorectal cancers the expression of Gal-8 is reduced, which also makes the prognosis of these patient’s poor (9-11).

Some studies have shown that Gal-8 has strong relationship with cell growth and also metastasizes in some human cancers, especially prostate cancer. Also, in one study, the effects of this lectin on cell adhesion were studied due to the adhesion of cancerous cells to vascular endothelium, and given that Gal-8 serum levels in patients with colon cancer and breast cancer had increased (12). 

Gal-8 is also an angiogenesis regulator in the endothelium of blood and lymphatic vessels that can detect different ligands in both types of endothelium. Gal-8 is actually expressed in both of cytoplasm and nucleus of endothelial cells of the vessels of normal and neoplastic tissues, in which it plays an important role in the in vivo development of new vessels (angiogenesis) and the migration of endothelial cells. This issue can be a debatable topic for the treatment of cancerous lesions or preventing their metastasis. (13)

Thus, studies have shown over the years that there is a link between galectin expression and the progression of tumors as well as their metastasis and today it can be said that these proteins can also be used as tumor markers (1, 14,15). 

Oropharyngeal and laryngeal cancers are ranked as the sixth most common cancer in the world; it is estimated that 550,000 people annually are involved with these cancers and about 300,000 of them die (16). The prevalence of these diseases is increasing with the increase in smoking and alcohol use worldwide (17). Gal-8 is a glycan-binding protein that play a lot of roles in a variety of biologic processes such as cell protection from injurious stimuli, modulate autoimmunity, antimicrobial effect, cell-cell and cell-matrix interaction, in cell proliferation, cell death, or even cell migration (3,9,13, 18-22). Although the usefulness of Gal-8 has been partly studied in the past, the results about significance of this marker in tumorigenesis of various organs are different and occasionally controversial (7-9,15-17); in addition, its role in head and neck squamous cell carcinoma (SCC) has not been fully identified.

Therefore, the roles of this protein in the carcinogenic pathway of SCC, the predominant histologic type of head & neck carcinoma, maybe crucial, and we are going to examine the expression of Gal-8 in normal, dysplastic and malignant squamous epithelium of head and neck by IHC technique. It may be possible, through this biomarker, to take a new and important step in the early diagnosis of precancerous conditions versus to atypical regenerative hyperplasia, treatment and even prevention of metastasis of head & neck SCC.

## Materials and Methods

This is a retrospective cross-sectional descriptive and analytical study in which all patients with a diagnosis of SCC or dysplasia of the head and neck, who underwent biopsy or surgery before adjuvant therapy from 2015 till 2017 in Boo-Ali SINA hospital of Sari, Iran, were chosen by census method. Subjects with history of chemotherapy, radiotherapy and steroid consumption before sampling and those with uncertain diagnosis were excluded. The sample size was 93 including normal, dysplastic and tumor tissues. At first, the required information including age, sex, tumor location, the degree of tumor differentiation and tumor stage were extracted manually from patients pathology report and from medical records center of Boo-Ali SINA hospital, the obtained data were noted on questionnaire of the proposal. All relevant tissues that were fixed in 10% formalin and embedded in paraffin were collected from pathology archive. All cuts of blocks that were located in the tumor site and the normal tissue that was as far as possible adjacent to it were examined. The presence of cancers was confirmed by review of the slides to enter the study, and cuts that contained appropriate tumoral tissue were obtained for immunohistochemistry(IHC) staining. 

This study was approved by the Vice-Chancellor of Research and Technology of Mazandaran University of Medical Sciences (grant:4925) and by Ethics Committee in Biomedical Research (IR.MAZUM.REC.1397.360). During all stages of this study, we adhered to the Helsinki provisions. 

**Immunohistochemical Staining:**

The following steps were executed for staining: At first Paraffin removal from paraffin block samples were done, then samples were dehydrated by ascent concentrations of ethanol (77%, 95% and 97%) and placement in TRIS-EDTA buffer (pH = 9). Then clarifying the samples by xylene and tissue cutting with thickness of 3-4 μm was performed respectively. Then transferred to the microwave; buffer was then added the and was allowed to reach the boiling point, then the microwave power dropped to 40%, and after 15 minutes, the tissues were removed and placed at room temperature for 15 minutes. After washing with water and TBS buffer, using a DAKO pen, the tissue was identified and placed in a humid chamber. After that’ it was treated by a human polyclonal antibody diagnostic kit according to manufacturer’s recommendations (Anti-Galectin 8,100 μg, Elabscience Co., USA), the antibody diluted to 1/500 dilution with antibody diluent and then the conjugates were incubated at room temperature overnight. Then the tissues were placed in the vicinity to peroxidase for 30 minutes and then the chromogenic reaction was performed by the appropriate kit. In the end, samples were placed in the room for 30 minutes. After washing with TBS buffer and completing the IHC and hematoxylin staining steps as background color, slides were prepared.

**Immunohistochemical Evaluation:**

A semi-quantitative examination was performed by two independent pathologists, in single-blind study without knowledge of patient's data, using an optical microscope, in low and high-power fields of slides based on the mean of estimated percentage of stained tumor cells as well as intensity of staining (23).

The samples were classified in terms of the percentage of stained of the cells: in the case of non-stained, the score 0, in less than 10% the score of 1, between 10 to 50% the score of 2 and more than 50% the score of 3 was considered. Also, intensity of staining was scored 0 (no), 1(weak), 2(moderate) and 3 (strong). Then the total score was calculated by summation scores of percentage and intensity of cytoplasmic /nuclear staining, if scored 2 or less, it was considered negative and if scored greater than 2, considered positive (23).

**Data Analysis**

Data were entered into SPSS version 22 (SPSS Inc., Chicago, Ill., USA). Descriptive statistics for quantitative variables and frequency tables for qualitative variables were used to describe the data. Frequency percentage of qualitative variables was calculated and compared with the McNemar test. To compare quantitative variables paired t-test was used. The level of P-value<0.05 was considered significant.

## Results

As showen by Table 1, 81.7% of the patients aged over 50 years old, 58.1% were male and remaining were female. The most frequent site of sampling was oral cavity.

Overall, 44.1% of the selected paraffin blocks were normal tissue and 14% were dysplastic and 41.9% were malignant. 

The study of age status in the three diagnostic groups at the error level of 0.05 according to Fischer's exact test did not show meaningful difference and the groups were homogeneous in terms of age distribution. The analysis of sex status and location of the tumor showed that there was no significant difference between the three diagnostic groups at the level of 0.05 by the Chi-square test and the groups were homogeneous in terms of gender and site distribution (Table 2).

The pathologic features of tumor in the cancer patients, is shown by Table 3. 

The IHC results showed that in the normal group, 0%, 2.4%, 51.2%, and 46.3% had zero, one, two and three scores in staining intensity, respectively. In the dysplastic group, the findings showed that 23.1%, 38.5%, 30.8%, and 7.7%, respectively, had zero, one, two, and three scores. In the cancerous group, 17.9%, 48.7%, 33.3% and 0% of the samples, had zero, one, two, and three scores, respectively.

Also, on the other hand, among the samples with a zero score of stain intensity, 0% were normal, 30% were dysplastic and 70% were cancerous. Among those who had one score, 4% were normal, 20% were dysplastic and 76% were cancerous. Among those who had two scores of staining level, 55.3% were normal, 10.5% were dysplastic and 34.2% were cancerous and among those that had three scores, 95% were normal, 5% were dysplastic and 0% was cancerous.

Evaluation of the percentage of the stained cells showed that in the normal group, 0%, 2.4%, 9.8%, and 97.8% cases had zero, one, two and three scores respectively. In the dysplastic group, the findings showed that 15.4%, 38.5%, 30.8%, and 15.4%, respectively, had zero, one, two, and three scores. In the cancerous group, 17.9%, 51.3%, 20.5% and 10.3% of the samples were scored zero, one, two, and three, respectively.

In addition, among the samples with unstained cells, 0% were normal, 22.2% were dysplastic and 77.8% were cancerous. Among those who had one score ,3.8% were normal, 19.2% were dysplastic and 76.9% were cancerous. Among those who had two scores, 25% were normal, 25% were dysplastic and 50% were cancerous. Between cases with score 3, 85.7% were normal, 4.8% were dysplastic and 9.5% were cancerous. Results showed that the intensity and percentage of staining depended on the subjects’ pathologic group (*P*=0.0001) (Table 4). The expression of gale-8 did not correlate with stage (*P*=0.303), lymph node involvement (*P*=0.326), tumor grade (*P*=0.769), distant metastasis (*P*=0.748), and age (*P*=0.574). 

**Fig 1:**
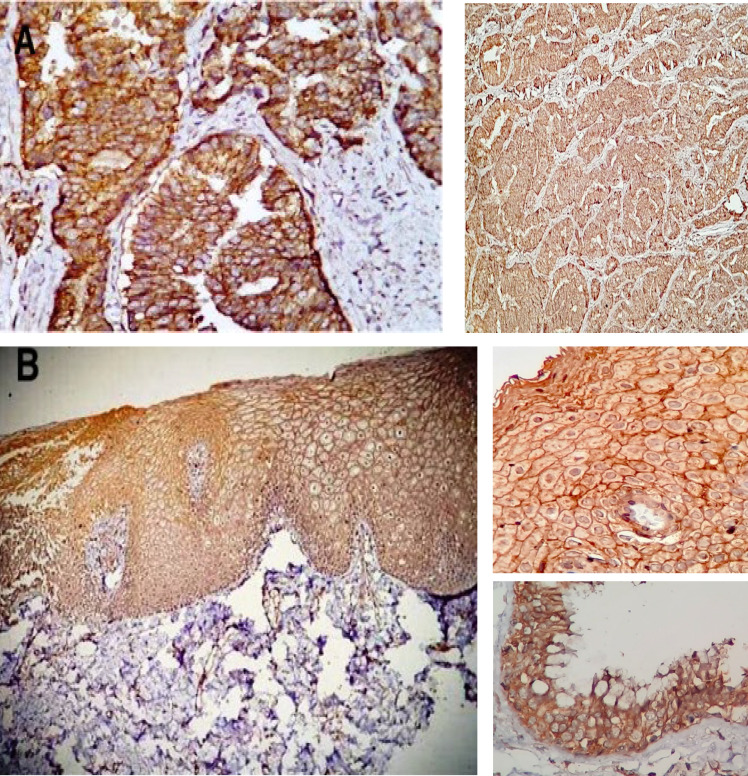
Illustrated IHC stained slides with Anti galectin-8 antibody, under light microscope, revealed: A- Positive prostatic adenocarcinoma as control sample, that neoplastic glands colored brown and the stroma-colored violet, by 400× magnification. B- Normal squamous epithelium and its strong positive cytoplasmic staining that merging to cell membrane. also, good staining of the cell nuclei is in evident, by 200×magnification.text box shows high power view of squamous epithelium and respiratory epithelium

**Fig 2:**
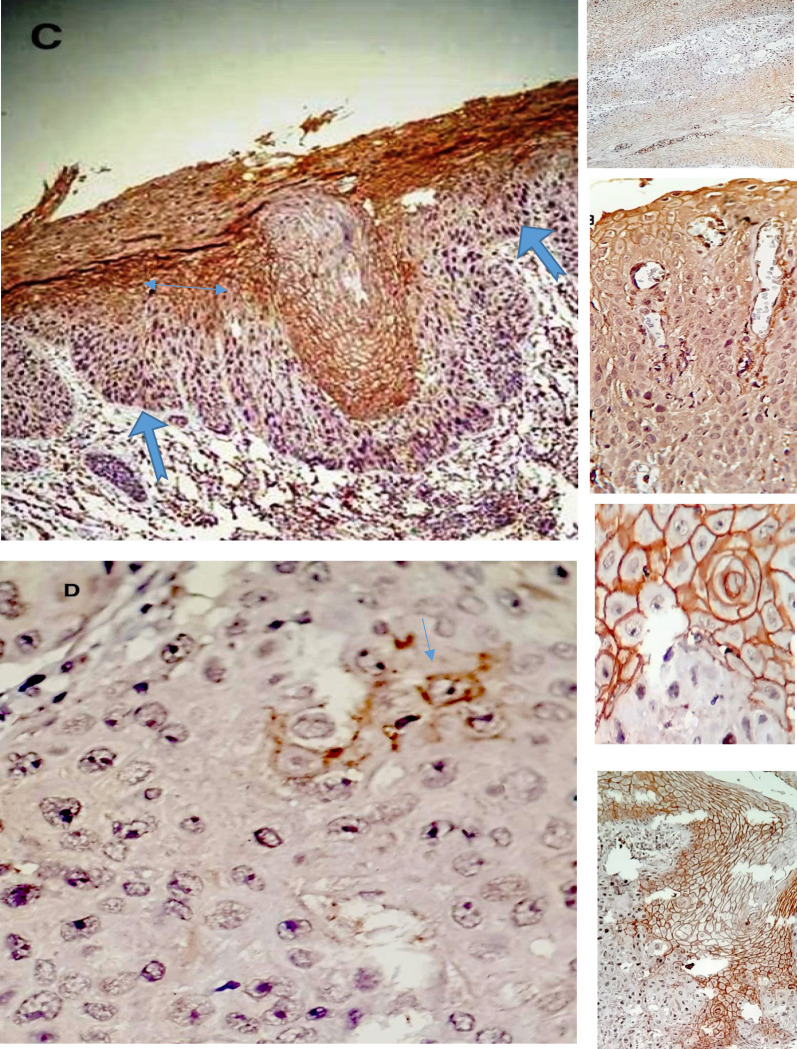
Illustrated IHC stained slides with Anti galectin-8 antibody, under light microscope, showed: C- Reduced staining in dysplastic areas (notched arrow), contrasting to, nondysplastic upper third part of the epithelium (double arrow) that stained brown, by 200× magnification. text box shows gradual reduction of coloring from spinous layer to basal layer. D- Marked Reduction of staining in the SCC. Focal, heterogenous and weak positive cytoplasmic staining (arrow) in some of the neoplastic squamous cell is noted. No nuclear staining has been shown, by 400× magnification. text box shows interface of tumor with its adjacent nontumoral area

According to above mentioned criteria in the Method section, 97.6% of the examined normal tissues revealed moderate to strong uniform membranous and nuclear positive staining with galectin-8 (Figure 1), while 77% of dysplastic lesions and 69% of the cancers revealed negative immunoreactivity. The positive staining pattern in the dysplastic and malignant areas were patchy, variegated and often restricted to cell membrane (Figure 2) 

**Table 1:** Pathological characteristics of samples

		Frequency	%
Site of sampling	Oral cavity	47	**49.6**
	Nasopharynx	4	**4.3**
	Hypopharynx	2	**2.2**
	Larynx	35	**37.6**
	Scalp and neck skin	5	**5.4**
Result of pathologic evaluation	Normal tissues	41	**44.1%**
	Dysplasia	13	**14.0**
	SCC	38	**40.8**
**Nasopharyngeal carcinoma**	**1**	**1.1**

**Table 2:** Evaluation of frequency distribution of clinical characteristics of patients

		Normal			Pre-cancerous			Cancerous			**P-value**
		N	Row%	Column%	N	Row%	Column%	N	Row%	Column%	
Age	**<50 yrs.**	32	42.1	78.0	12	15.8	92.3	32	42.1	82.1	**0.577**
	**>50 yrs.**	9	52.9	22.0	1	5.9	7.7	7	41.2	17.9	
Gender	**male**	23	42.6	56.1	7	13.0	53.8	24	44.4	61.5	**0.838**
	**female**	18	46.2	43.9	6	15.4	46.2	15	38.5	38.5	
Site of sampling	**oral cavity**	23	50.0	56.1	5	13.6	23.1	19	36.4	48.6	**0.690**
	**nasopharynx**	1	25.0	2.4	0	0.0	0.0	3	75.0	7.7	
	**hypopharynx**	0	0.0	0.0	1	50.0	7.7	1	50.0	2.6	
	**larynx**	15	42.9	36.6	5	14.3	38.5	15	42.9	38.5	
	scalp and neck	2	40.0	4.9	2	40.0	15.4	1	20.0	2.6	

**Table 3:** Evaluation of pathologic parameters of head and neck SCC

Variable		Frequency	%
Tumor histologic grade	low	22	**56.4**
	Moderate	12	**30.8**
	high	3	**7.7**
	undifferentiated	2	**5.1**
Lymph node involvement	Yes	4	**10.2**
	no	17	**43.6**
	unspecified	18	**46.2**
Distant metastasis	Yes	0	**0.0**
	no	21	**53.8**
	unspecified	18	**46.2**
Stage of tumor	unspecified	18	**46.2**
	1	10	**25.6**
	2	7	**17.9**
	3	2	**5.1**
	4	2	**5.1**

**Table 4:** Determination and comparison of intensity and percentage of staining in three groups

		Normal			Pre-cancerous			Cancerous			P-value
		N	Row%	Column%	N	Row%	Column%	N	Row%	Column%	
Intensity	**Negative**	0	0.0	0.0	3	30.0	23.1	7	70.0	17.9	**0.0001**
	**Weak**	1	4.0	2.4	5	20.0	38.5	19	76.0	48.7	
	**Moderate**	21	55.3	51.2	4	10.5	30.8	13	34.2	33.3	
	**Strong**	19	95.0	46.3	1	5.0	7.7	0	0.0	0.0	
Percentage	**0**	0	0.0	0.0	2	22.2	15.4	7	77.8	17.9	**0.0001**
	**1**	1	3.8	2.4	5	19.2	38.5	20	76.9	51.3	
	**2**	4	25.0	9.8	4	25.0	30.8	8	50.0	20.5	
	3	36	85.7	87.8	2	4.8	15.4	4	9.5	10.3	

## Discussion

Gal-8 was discovered in 1995 from mouse liver cDNA and has been recently a subject interest for researchers. To the best of our knowledge, there is limited study about potential roles of Gal-8 in the carcinogenesis of head and neck SCC, so the aim of this study was investigating the expression of Gal-8 in normal, dysplastic and malignant squamous epithelium of head and neck by immunohistochemistry. Results of this work showed that the intensity and percentage of staining depended on the subjects’ pathologic group (*P*=0.0001).

Zick *et al.* investigated immunohistochemical expression of Gal-8 in human colon tissue samples. They found Gal-8 markedly decreased in cancer compared with normal and dysplastic mucosa of colon and inversely correlated with cancer invasiveness. Furthermore, they assessed the role of this marker in the migration of different colon cancer cell lines and assumed that it acts as a potent suppressor tumor growth rate. Their results were concordant with our observations on the Gal-8 expression in human head and neck SCCs, which was significantly lower than normal squamous epithelium (5).

A study by Dong *et al.* with immunohistochemical analysis showed that there is a strong positive association between Gal-8 and the disease stage of laryngeal SCCs, which was not compliant with our observation. However, the presence of malignancy was inversely correlated with expression of Gal-8 in SCC of the larynx; they finally stated that the expression of the Gal-8 could be used as a prognostic factor for Laryngeal SCCs (14).

Cludts *et al.* examined Gal-8 as a progression factor in the laryngeal tumor and compared it with galectins-1, -3 and -7. In this study, expression of Gal-8 was evaluated by immunohistochemistry technique in 18 and 16 cases of normal epithelium, 24 and 10 cases of low-grade dysplasia, and 22 and 15 cases of high-grade dysplasia around the tumor, and 74 and 37 cases of hypopharyngeal and laryngeal cancer respectively. It turned out increasing the intensity of Gal-8 staining and the greater extent of the immunopositivity in malignancy were seen against dysplasia in hypopharyngeal cancer and in laryngeal cancer. However, they did not show any association with recurrence. They ultimately stated that the presented data showed a divergence in the galectins-1, -3, -7, and 8 networks during tumor progression (17). In the present study, both the intensity and staining of the Gal-8 decreased in cancerous tissue. The disparate results observed in the analysis of Gal-8 by Cludts and our study might be due to different antibodies that were used for IHC or might be due to diversity in the mutations of the LGALS8 gene among populations.

In a review study, Wagner *et al.* examined the role of Gal-8 in various types of cancers. They reported that Gal-8 protein is expressed in the tumor tissue and in the normal tissue. The level of expression of Gal-8 is associated with malignant colon cancer and differentiation of SCC and neuromuscular tumors of the lung. Recently, the difference in the rate of expression of this marker has been used for therapeutic strategies in lung SCC, suggesting that usefulness of this marker is still unknown in other cancers, and it is necessary to examine its role in other cancers (24). 

Another review in 2001 by Kiss *et al.* on the expression of Gal-8 in benign and malignant epithelial, mesenchymal and even neurological tumors in different parts of the body showed decrease in expression of Gal-8 in malignant tumors compared with normal and benign tumors in colon (*P*=0.001), pancreas (*P*=0.007), liver (*P*=0.0008), skin (*P*=0.002) and larynx (*P*=0.02), respectively. Furthermore, in this study, the normal tissue of the oropharynx compared to cancer and dysplasia had more Gal-8 stain intensity, which is similar to the results of our study. An excess amount of this marker was observed in breast cancer. In the Kiss study, there was no difference in staining of the bladder, kidney, lung, prostate and stomach in normal tissue with benign and malignant tumors (25). 

A study by Gentilini *et al.* declared that Gal-8 expression can predict the metastatic potential of a malignant prostate tumor. They found a high Gal-8 expression rate in the well differentiated prostatic adenocarcinoma compared with normal prostate might delay the cancer progression. By means, low or lack of Gal-8 expression in high grade prostatic adenocarcinoma might directly lead to a worse outcome (26).

A review article by Hossaka *et al.* that explains the roles of galectins in oral cavity cancers, with a focus on studies about galectins 1 and 3 in this area, recommends further studies on the molecular pathway to find new therapies (27). Their advice would be also rational for Gal-8 investigation in the head & neck carcinogenesis.

Gurung *et al.* noted that Gal-8 was highly expressed in papillary thyroid cancer in comparison with normal thyroid gland by IHC and Real-time PCR methods (*P*<0.0001). Gal-8 had 97% sensitivity and 62% specificity for this cancer. They noted that, the Gal-8 staining pattern has changed in malignant cells with more tendency to cell nuclei (23). In line with their study, we saw alteration in the normal Gal-8 staining pattern in dysplastic and neoplastic cells, however we observed patchy and weak cytoplasmic coloring without notable staining of the cell nuclei. This discrepancy can be attributed to variety in biologic roles of Gal-8 in these two differed human anatomic sites. 

Meinohl *et al.* by mass spectrometry analyses and protein interaction assay declared that Gal-8 binds to oncogenic K-Ras4B via farnesylation of its c-terminus, thereby modulates cellular migration and proliferation in lung and pancreatic carcinoma cell lines. They experimented that, depletion of Gal-8 mediated by siRNA, made interferences with intracellular K-Ras4B ERK1/2 signaling pathway and inhibited cell growth. They proposed the inhibition of Gal-8 may provide an opportunity to prevent cancer progress since this marker is overexpressed in carcinoma that doesn’t match with our findings (28). 

Also, the presence of Gal-8 in lung tumor cells and the absence or low levels of it in normal lung tissue allows the use of monoclonal antibodies (Po66) for the treatment of lung cancer, in the beginning, this antibody was specifically able to connect to Gal-8 and was used to detect human SCC by immunoscintigraphy (8,9).

Duray *et al.* showed a decrease in expression of Gal-8 in sinonasal SCCs by lectin profiling that is in line with our project (29). Some researchers believe that downregulation of Gal-8 expression can promote the development of urothelial cancer. A significant difference was found comparing normal urothelium with any tumor stage (*P*<0.01) by Kramer *et al.* They concluded that loss of Gal-8 might be an early step in the malignant transformation of the bladder urothelium and is an independent predictor of its recurrence (30).

 Kindt *et al.* reviewed the roles of galectins in carcinogenesis of different organs especially head and neck SCC and thyroid cancer. They noted Gal-8 was highly expressed in the cytoplasm of normal ductal cells of salivary glands, but their malignant cells often showed low level of cytoplasmic reactivity with this marker and occasionally reacted with it in the nucleus (31).

Labrie *et al.* compared the plasma levels of Gal-8 in 145 cases of ovarian cancer with its level in 160 healthy women, they found, a high level of Gal-8 in high-grade serous cancer moreover pointed this finding predict a poor outcome. They concluded that measurement plasma levels of Gal-8 has a potential to predict prognosis of high-grade serous cancer of the ovary. Although we did not check plasma levels of Gal-8, its utility in head & neck SCC is indicative to be tested. In addition, they examined normal samples of ovarian and fallopian tube tissue for Gal-8 expression by immunofluorescence method, they detected Gal-8 in the cytoplasm of epithelial cells which is consistent with our observation about normal head & neck epithelium (22). 

 Remmelink *et al.* tested a panel of galectins in different tumors of the salivary gland by IHC, and found diversity in expression pattern of the galectins among them, and assumed this finding may be beneficial in distinction of these neoplasms. They pointed out a special pattern of Gal‐1, -3, -7 and -8 expression that was restricted to cytoplasm of intermediate cells in mucoepidermoid carcinomas (32). In line with their study, we observed that analysis of Gal-8 by IHC has a potential guide to distinction of benign/reactive squamous cells from dysplastic/malignant squamous cells in the upper aerodigestive system.

In summary, we observed strong and diffuse expression of Gal-8 in the entire circumferences of the cell membrane of normal squamous epithelium of head and neck. We postulated that this protein may contribute to preserve normal polarity and connection of squamous epithelial cells. In our opinion, weak expression or lack of this bonding protein may disrupt intercellular convergence, thereby may facilitate malignant transformation of head and neck squamous epithelium.

## Limitation and Suggestion

We acknowledge that our sample size was small and among cancer, distant metastases' status was frequently unclear, these problems may be the reason of the non-significant relationship between Gal-8 and disease stage. Future or pooled studies are required to verify its value for prediction of tumor behavior.

## Conclusion

This study showed the expression of Gal-8 in dysplastic and cancerous tissues is lower than normal squamous epithelium of head and neck. We propose that dysregulation of Gal-8 may contribute to the development of head and neck SCC.
